# Colloidal CsPbX_3_ Nanocrystals with Thin Metal Oxide Gel Coatings

**DOI:** 10.1021/acs.chemmater.2c03562

**Published:** 2023-03-20

**Authors:** Dominic Guggisberg, Sergii Yakunin, Christoph Neff, Marcel Aebli, Detlef Günther, Maksym V. Kovalenko, Dmitry N. Dirin

**Affiliations:** † Department of Chemistry and Applied Biosciences, ETH Zürich, Zürich CH-8093, Switzerland; ‡ Laboratory for Thin Films and Photovoltaics, Empa - Swiss Federal Laboratories for Materials Science and Technology, Dübendorf CH-8600, Switzerland; § NCCR Catalysis, Institute of Inorganic Chemistry, Department of Chemistry and Applied Biosciences, ETH Zürich, Zürich CH-8093, Switzerland

## Abstract

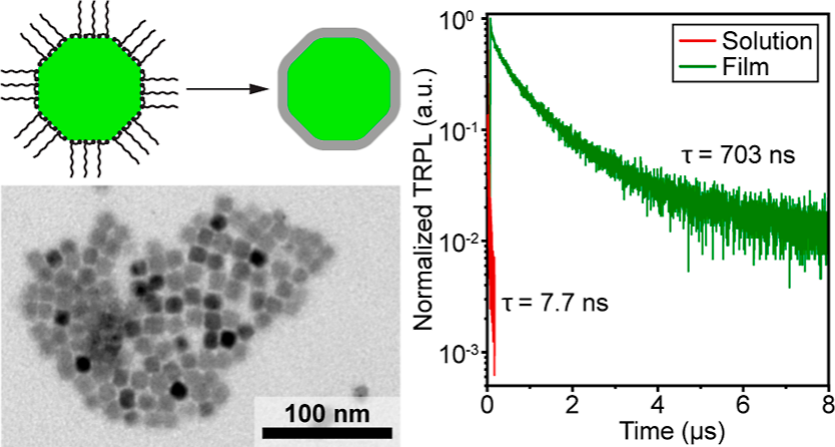

Lead halide perovskite (LHP) nanocrystals (NCs) have gathered much attention as light-emitting materials, particularly owing to their excellent color purity, band gap tunability, high photoluminescence quantum yield (PLQY), low cost, and scalable synthesis. To enhance the stability of LHP NCs, bulky strongly bound organic ligands are commonly employed, which counteract the extraction of charge carriers from the NCs and hinder their use as photoconductive materials and photocatalysts. Replacing these ligands with a thin coating is a complex challenge due to the highly dynamic ionic lattice, which is vulnerable to the commonly employed coating precursors and solvents. In this work, we demonstrate thin (<1 nm) metal oxide gel coatings through non-hydrolytic sol–gel reactions. The coated NCs are readily dispersible and highly stable in short-chain alcohols while remaining monodisperse and exhibiting high PLQY (70–90%). We show the successful coating of NCs in a wide range of sizes (5–14 nm) and halide compositions. Alumina-gel-coated NCs were chosen for an in-depth analysis, and the versatility of the approach is demonstrated by employing zirconia- and titania-based coatings. Compact films of the alumina-gel-coated NCs exhibit electronic and excitonic coupling between the NCs, leading to two orders of magnitude longer photoluminescence lifetimes (400–700 ns) compared to NCs in solution or their organically capped counterparts. This makes these NCs highly suited for applications where charge carrier delocalization or extraction is essential for performance.

## Introduction

Lead halide perovskite (LHP) nanocrystals (NCs) have emerged as promising light-emitting materials^1−3^ for liquid crystal displays,^4−7^ LEDs,^8−11^ lasers,^12−14^ scintillators,^15−17^ luminescent solar concentrators.^18−20^ and as quantum emitters.^21−24^ They combine broadly tunable emission colors (405–770 nm)^25,26^ with high photoluminescence quantum yield (PLQY)^25−27^ and PL linewidths as narrow as 70 meV, enabling exceptional color purity.^28^ Recently, LHP NCs have also been considered as photocatalysts^29−38^ or photoconductive materials.^39,40^ These applications would greatly benefit from surface accessibility and energy transfer capabilities.^33−35,41−43^

While the commonly employed long-chain organic ligands provide surface passivation and render NCs colloidally stable, they also act as an insulating barrier. Smaller ligands, especially inorganic ones, have shown to greatly improve the charge transport in nanocrystal solids.^44,45^ Unfortunately, the choice of materials for inorganic coatings is severely limited due to the structurally labile lattice of LHPs.^2^ First, the coating material needs to be thermodynamically stable enough to protect the LHP core from any outside influence and prevent the diffusion of the highly mobile halide anions out of the NCs. Second, among the stable materials, those that react with the LHP or employ precursors reactive toward the LHP need to be excluded as well. This eliminates most halide and chalcogenide salts since they lead to anion exchange or extraction of the Pb to form a more stable compound (PbF_2_, PbS, PbSe, etc.). Third, the coating should not introduce trap states or significantly alter the optical properties. The obvious choice that remained for us was oxide-based coatings. Recently, many groups have focused on encapsulating LHP NCs in an oxide-based inorganic matrix^46−49^ or tried to synthesize stable LHP core/inorganic shell NCs.^3,50−52^ A vast majority of these publications are based on SiO_2_ and related oxides, showing that they are indeed chemically inert toward the LHP and that the optical properties of coated NCs remain virtually unchanged.^3,51,53^ However, most of these works focus on rather thick coatings in an attempt to create fully environmentally stable LHP compounds. On the contrary, in this work, we sought to realize colloidally and environmentally stable LHP NCs coated with a thin inorganic layer and free of bulky organic ligands.

Besides the coating material, the synthesis path must be judiciously chosen too. A common theme among the published oxide coatings for LHP NCs is the hydrolytic sol–gel reaction used for synthesis. Hence, the synthesis outcome is a compromise between the rate of NC degradation and shell formation. Buonsanti et al. proposed a work-around by using an aluminum precursor that reacts with molecular oxygen to give a mixed alumina-organic ligand coating.^57^ To our knowledge, a non-hydrolytic sol gel reaction has thus far not been reported for coating LHP NCs. Herein, we present such a path, yielding a sub-nm thin oxide gel coating. The obtained NCs proved to be more stable toward polar organic solvents than their organically capped counterparts while only being covered by a thin gel layer. We showcase alumina-, titania-, and zirconia-based coatings to demonstrate the versatility of this approach. Compact films of such NCs feature strong delocalization of the photoexcited carriers, leading to PL lifetimes two orders of magnitude longer (400–700 ns) than their organically capped counterparts, opening an avenue for applications where strong electronic coupling of individual NCs is essential.

## Results and Discussion

**Figure 1:**
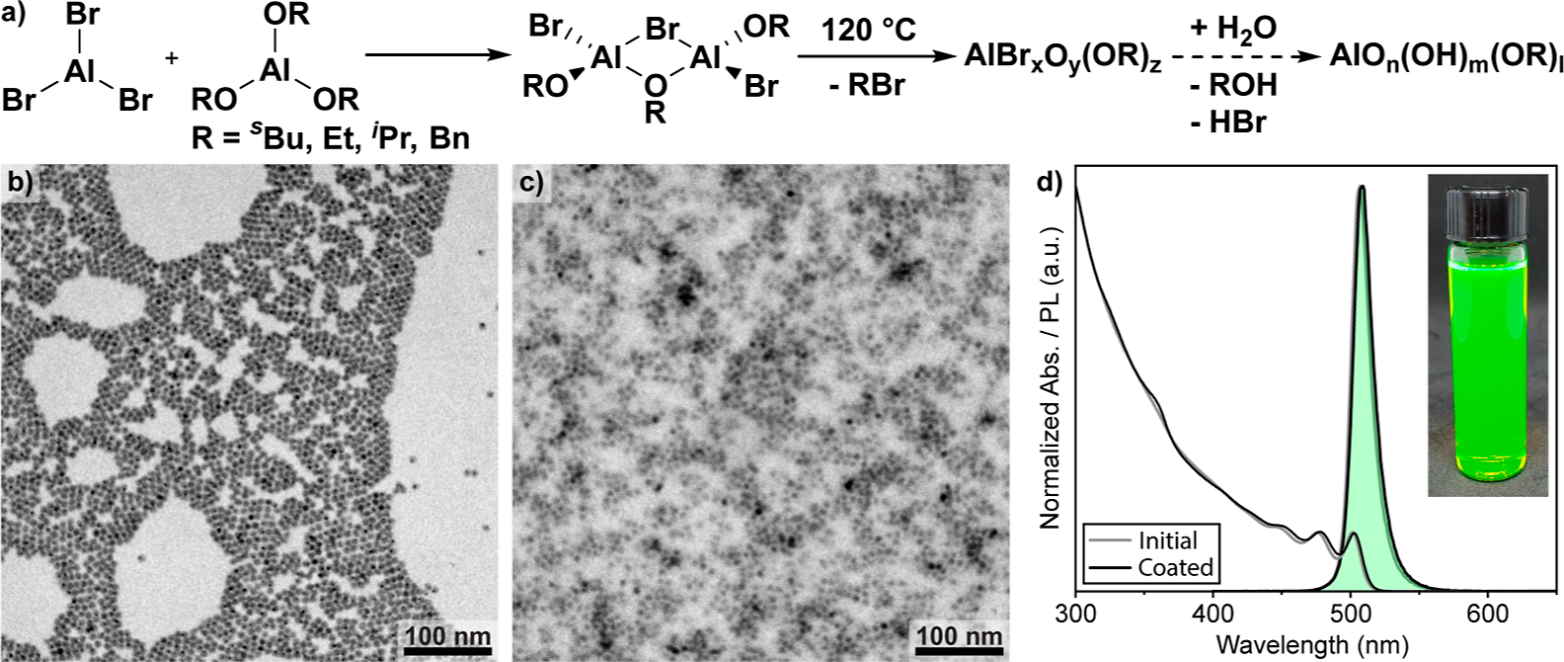
(a) Reaction scheme for the formation of an alumina gel by condensation of aluminum bromide and an aluminum alkoxide via alkyl halide elimination. An alumina gel is produced after heating to 120 °C and is capable of further hydrolyzing when exposed to ambient humidity.^54−56^ (b,c) TEM images of zwitterion-capped and alumina-gel-coated CsPbBr_3_ NCs. (d) Normalized absorption and emission spectra of the initial zwitterion-capped NCs (gray lines) and the alumina-gel-coated NCs (black lines), with a representative image of alumina-gel-coated CsPbBr_3_ NCs dispersed in *n*-butanol.

Alumina-gel-coated LHP NCs were obtained by first synthesizing organically capped NCs (Figure 1b) using a scaled-up synthesis previously reported by our group (synthesis details in the Supporting Information).^58^ CsPbBr_3_ NCs capped with 3-(*N*,*N*-dimethyloctadecylammonio)propanesulfonate (ASC18), a zwitterionic ligand introduced in our earlier work,^59^ were determined to work best for the subsequent alumina gel coating. The commonly employed oleic acid/oleylamine ligand system^25^ did not sufficiently protect the NCs during the coating step, and the NCs sintered and degraded before a sufficient amount of coating material was deposited. A similar outcome was observed for other ligands; for example, phosphatidylcholine-based ligands like lecithin^60^ degraded when heated and consequently were not capable of protecting the NCs. In the second step, the sol–gel coating was formed by reacting an aluminum halide AlX_3_ (X = Cl, Br, and I) and an aluminum alkoxide Al(OR)_3_ (R = Et, ^*s*^Bu, ^*t*^Bu, and Ph) in the presence of NCs, as outlined in Figure 1a.^54−56^ Particularly, ASC18-capped CsPbBr_3_ NCs (0.12 mmol) in toluene were mixed with ODE (12 mL) and dried under vacuum. A solution of AlBr_3_ (0.18 mmol) and Al(O^*s*^Bu)_3_ (0.18 mmol) in anhydrous mesitylene (1 mL) was prepared in the glovebox and injected into the NC colloid at room temperature under vigorous stirring. The solution was subsequently heated to 120 °C for 10 min and then cooled to room temperature with a water bath. Coated NCs were precipitated from the crude solution with acetone (24 mL), leading to a final hydrolysis step of the gel with present trace amounts of water. This also introduced surface charges, and the NCs were now dispersible in short-chain alcohols such as ethanol, isopropanol, or *n*-butanol. Particularly, in *n*-butanol, high concentrations (up to 100 mg/mL of LHP) could be obtained. An excess alumina gel is produced, which displaces the zwitterionic ligands on the surface. The amount of aluminum precursors is adjusted such that the formation of free gel is minimized. When formed, the excess gel can either be kept for additional protection, in particular, when producing films, or be washed away. Alumina-gel-coated NCs can be precipitated multiple times with diethyl ether or hexane and re-dispersed in the solvent of choice. This gradually reduces the amount of free gel in solution until a clean NC dispersion is obtained after 3 to 5 washing cycles. Typical transmission electron microscopy (TEM) images show fairly uniform perovskite NCs with a faint, low-contrast border attributed to the alumina gel coating (Figure 1c). Well-purified samples generally display very thin coatings (on the order of 1 nm) that are difficult to image even with a high-resolution TEM. In less purified samples, NCs with thicker coatings (up to 5 nm) can be found (Figure S1). The final NC dispersions show narrow emission (FWHM <20 nm) between 490 and 515 nm (depending on the size of NCs) with a PLQY of 70 to 90% (Figure 1d).

**Figure 2:**
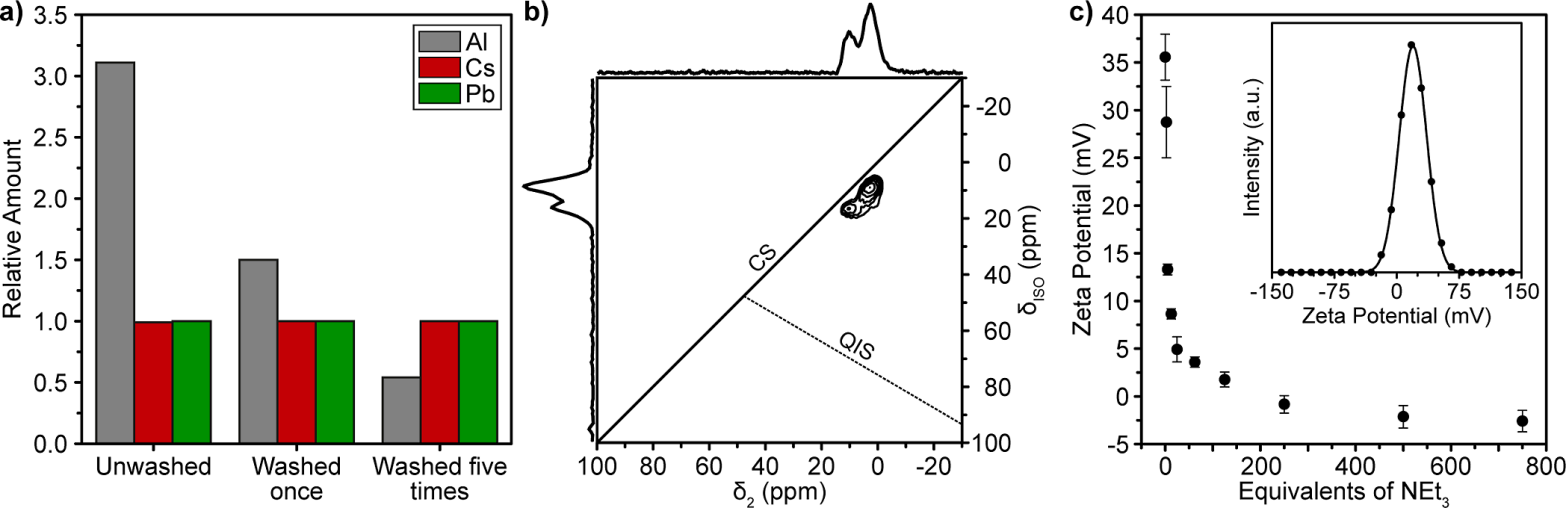
(a) Elemental composition of alumina-gel-coated NCs as derived from ICP–MS elemental analysis, normalized to the amount of Pb. Samples were washed to three different degrees without loss of colloidal stability or change in optical properties. (b) Solid-state ^27^Al MQMAS NMR spectrum of alumina-gel-coated NCs. Two distinct 6-fold coordinated AlO_*x*_ species can be observed.^54^ (c) Change in the ζ potential of alumina-gel-coated NCs when adding the base triethylamine. The inset is a representative ζ potential measurement of alumina-gel-coated NCs in butanol where no base was added.

Colloidal stability in alcohols evidences a surface modification upon sol–gel reaction, although the proof of an alumina gel coating is not straightforward. Inductively coupled plasma mass spectrometry (ICP–MS) elemental analysis yields the amount of Al-species that are tightly bound to the NCs and not removed during washing (Figure 2a). In this analysis, we compare a non-washed, a once-washed, and a five times-purified sample. All samples were precipitated with diethyl ether, dried under vacuum, and digested in a microwave with nitric acid. The ICP–MS measurement of the unwashed sample shows that all the aluminum put into the reaction could be recovered. Already, a single washing cycle removes approximately half of the aluminum from the sample, whereas the five times-purified sample had only 0.5 equiv of aluminum compared to lead remaining. Using this value, we determined the coating thickness to be less than 0.5 nm, corresponding to a mono-to-bilayer of aluminum-oxo species at most (calculation details in the Supporting Information). ^27^Al solid-state nuclear magnetic resonance (NMR) experiments were acquired to further characterize the alumina coating. A 1D spectrum of an alumina sol sample prepared without the addition of NCs shows a multitude of 4-, 5-, and 6-fold coordinated (at 65, 35, and 0 ppm, respectively) AlO_*x*_-species (Figure S2).^54^ Upon addition of NCs to the reaction, the undercoordinated aluminum species disappear. To increase the resolution, a multiple quantum magic-angle spinning (MQMAS) sequence^61^ was applied to an extensively washed NC sample (Figure 2b). This method separates the anisotropic quadrupole interaction from the isotropic chemical shift, thus allowing for a better identification of individual atomic environments, even in amorphous materials. Two distinct 6-fold coordinated species can be observed at 7 and 14 ppm, both exhibiting narrow peaks with 1 kHz FWHM and only a minor quadrupole-induced shift (QIS) of 4 ppm. These results show a high degree of symmetry around the aluminum atoms in alumina gel coating.

The alumina-gel-coated NCs are stabilized in solution via surface charge. To probe this charge, ζ potential (ZP) measurements were conducted. A potential of +30 to +40 mV was determined with no obvious trend on the amount of washing or coating thickness. This is on par with ζ potentials of aqueous Al_2_O_3_ NCs at pH 7.^62−64^ The charge in oxide systems stems from protonated/deprotonated oxo-species on the surface. As such, the charge is pH-dependent. The concept of pH does not directly translate to other solvents, but it should still be possible to deprotonate the NCs with a suitably strong base. Therefore, we added a controlled amount of triethylamine to NC solutions and found that the charge could be decreased to a minimum of about −4 mV (Figure 2c). When plotting the amount of base added on a log scale, the decrease in ZP becomes linear (Figure S5), similar to the linear dependence of ZP on pH that is often reported. Unfortunately, lower ZP values could not be measured due to the NCs starting to degrade under the measurement conditions when higher amounts of base were added.

**Figure 3:**
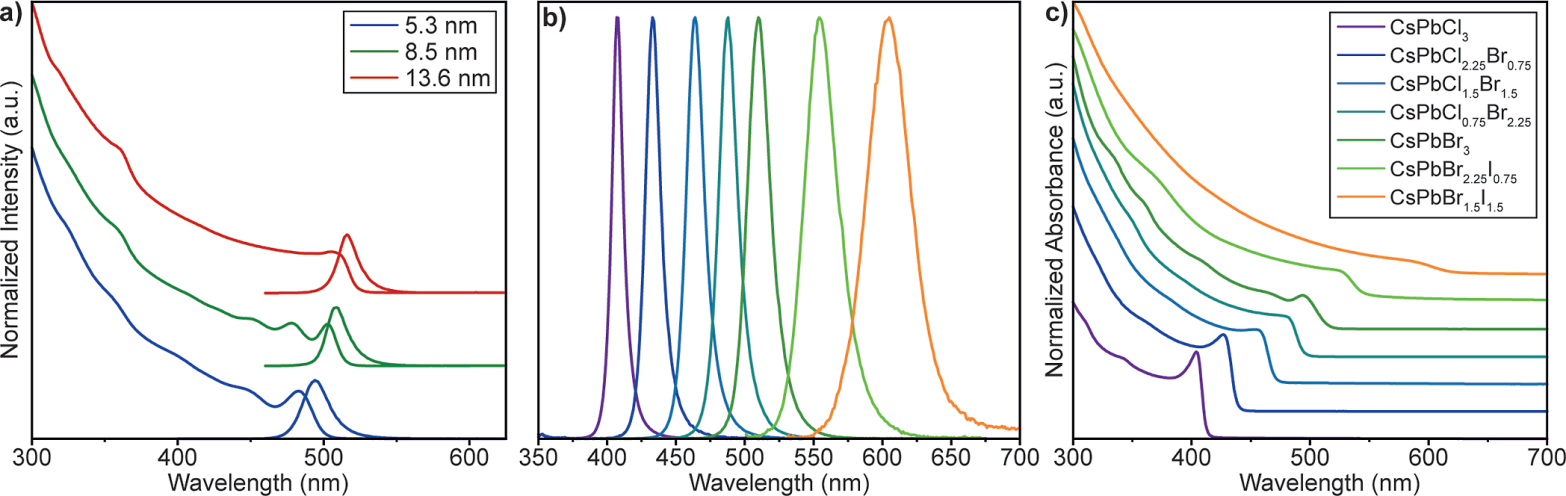
(a) Absorption and emission spectra of three different sizes of alumina-gel-coated NCs. (b,c) Normalized emission and the respective absorption spectra of alumina-gel-coated NCs with different compositions.

Three NC sizes were utilized to find that our coating approach is applicable for all sizes of NCs without compromising the NC’ integrity (Figure 3a). Since the coating obscures the effective core size in the TEM images, we used the maximum of the first absorption peak and the sizing curve published by Akkerman et al.^58^ to determine the core size. In all cases, the size increased only marginally from 4.6, 8.2, and 13.2 nm to 5.3, 8.5, and 13.6 nm, respectively.

LHP NCs feature PL that can be tuned throughout the whole visible spectrum by adjusting the halide composition.^25^ Since the coating procedure requires an aluminum halide, we adopted an approach wherein the coating and anion exchange^65^ occur simultaneously. A series of PL and absorption spectra covering the visible spectral range (Figure 3b,c) was obtained by varying the precursor ratios; AlCl_3_/AlBr_3_ (using CsPbCl_3_ or CsPbBr_3_ cores) or AlBr_3_/AlI_3_ (using CsPbBr_3_ cores). Successful coating was possible for any Cl–Br mixed composition. In the Br–I system, we could reach 80% iodine content, although the samples with more than 50% I were unstable. The polar sol–gel coating facilitated the expulsion of iodine from the NC core and promoted the transformation into the non-luminescent δ-phase^66,67^ upon dispersion in polar solvents. The thin labile gel coating does not prevent anion exchange between NCs of different compositions. In contrast, the unwashed samples coated with a weakly bound but thick gel layer exhibit several times slower anion exchange than the original organically capped NCs.^65^

**Figure 4:**
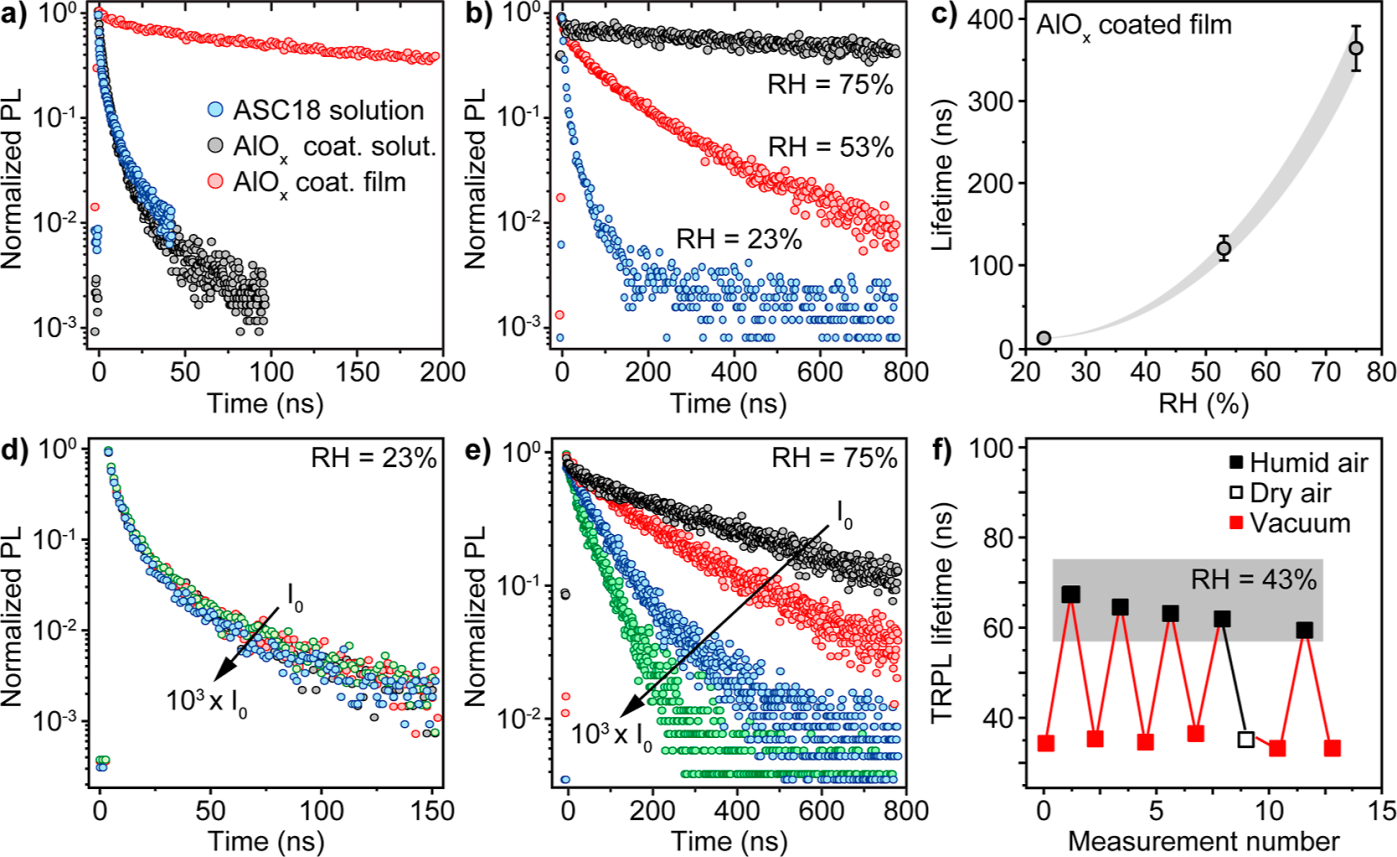
(a) TRPL traces for various NCs in solutions and film. (b) Humidity effect on TRPL traces for a film of alumina-gel-coated NCs. (c) Variation of TRPL lifetime with humidity. (d,e) Excitation beam intensity dependencies for TRPL traces for films of alumina-gel-coated NCs at RH = 23% (d) and RH = 75% (e). (f) Cycling of PL lifetime between the vacuum (red dots) or dry air (open black dot) and elevated humidity (filled black dots, RH = 43%—gray area) conditions.

Replacing the thick (∼2 nm) organic ligand shell with a thin (<0.5 nm) alumina gel coating is expected to enhance electronic and excitonic coupling of the NCs in a compact NC solid.^68,69^ When two or more semiconductor NCs are in close proximity to each other, their wave functions can couple to form delocalized states. This coupling manifests itself in a slight decrease in PL energy (Figure S8a) and a drastic extension of the time-resolved PL (TRPL) decay of alumina-gel-coated LHP NCs in compact film compared to the dilute solutions of the same NCs or analogous NCs capped with organic ligands (Figure 4a).

The efficiency of electronic coupling between NCs is defined not only by the distance between them but also by the height of the tunneling barrier. We found the second factor to be decisive in the case of NCs coated with the highly hydrophilic alumina gel. The TRPL lifetime of the compact NC solid changed by almost two orders of magnitude with an increase in relative humidity (R.H.) from 23 to 75% (Figure 4b,c). We hypothesize that adsorbed water increases the hydrolysis of the nanocrystal coating or raises its conductivity and thus enhances the excitonic and electronic coupling of the nanocrystals. Stronger coupling leads to delocalization of excitons and electrons and lengthening of the PL lifetime. Excitonic coupling in humid conditions and its insignificance in dry solid are also confirmed by TRPL dependences on pumping intensity. Compact NC solids prepared at RH = 23% show little change in TRPL lifetime when the excitation beam intensity is varied by three orders of magnitude (Figure 4d). Analogous NC solids exposed to humid conditions (RH = 75%) demonstrate strong acceleration of TRPL lifetime with increasing photon flux (Figure 4e). Note that the lifetime variations are largely reversible up to RH ∼60% and are caused solely by water vapor (Figures 4f and S8b). Elevated humidity also decreases PLQY in compact films due to the more efficient carrier delocalization, which increases the chance of non-radiative recombination. Specifically, films at 23% RH reach up to 75% PLQY, while at 75% RH, roughly 40% PLQY is observed (Figure S9).

**Figure 5:**
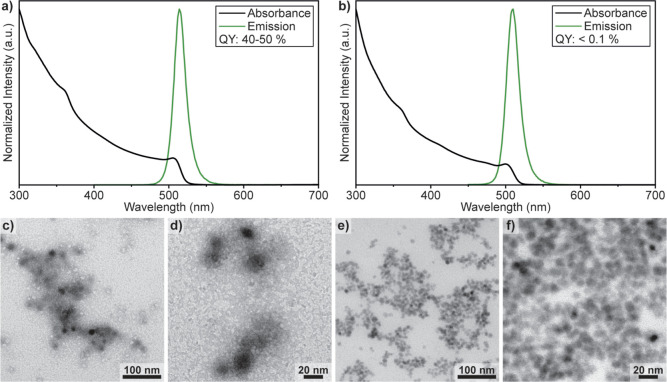
(a,b) Absorbance and emission spectra of zirconia- and titania-coated NCs, respectively. (c,d) TEM images of zirconia-coated NCs with excess zirconia sol visible around the NCs which cannot be removed to the same degree as for the other compositions. (e,f) TEM images of titania-coated NCs showing very uniform coatings.

In order to demonstrate the versatility of the non-hydrolytic sol–gel approach, we also synthesized zirconia- and titania-based analogues using ZrBr_4_ and Zr(OBu)_4_ or TiBr_4_ and Ti(O^*i*^Pr)_4_ precursors, respectively. Thin coatings were obtained in both cases which render the NCs stable in polar solvents. For the zirconia-coated NCs, the excess gel was difficult to wash away, and some of the leftover gel can be seen on the TEM images surrounding the NCs (Figure 5c,d). For titania, very clean and uniform coatings could be obtained (Figure 5e,f). Unfortunately, zirconia coatings seemed to be detrimental to the PLQY, and only 40–50% was achieved routinely (Figure 5a). Titania coatings severely quench the emission of LHP NCs to a level consistently lower than 0.1%, in full agreement with previous reports on TiO_2_ being an efficient electron scavenger for LHPs (Figure 5b).^70−73^ We note that the extraction of the photoexcited electrons from the conventional organically capped LHP NCs is known to be more challenging compared to the extraction of holes. Readily available and long-lived excited electrons in titania-coated LHP NCs are especially interesting for photocatalysis and open up new opportunities for LHP-based photocatalysts.

## Conclusions

We successfully coated LHP NCs with thin alumina-, zirconia-, and titania-based gels, which render NCs colloidally stable in short-chain alcohols. These coatings could be applied to NCs of any size in the range of 5–14 nm or halide composition without deterioration of the optical properties in the case of alumina gel coatings. Thin inorganic coatings make photoexcited carriers in LHP NCs more accessible, which is evidenced by electronic and excitonic coupling between NCs in a compact solid and opens up new opportunities for applications where carrier extraction or delocalization are essential.

## Experimental Methods

Full experimental procedures are provided in the Supporting Information.

### Alumina Gel-Coated CsPbBr_3_ NCs

In a typical synthesis, 0.12 mmol of the ASC18-capped NCs was mixed with ODE (12 mL), and the toluene was evaporated under vacuum. In the glovebox, a previously prepared solution of Al(O^*s*^Bu)_3_ (0.36 mL, 0.5 M in mesitylene, 0.18 mmol) was taken and mixed with a solution of AlBr_3_ (48 mg, 0.18 mmol) in 0.4 mL of anhydrous mesitylene. This results in roughly 0.8 mL of Al_2_Br_3_(O^*s*^Bu)_3_ precursor solution, which is injected into the NC solution at room temperature under continuous stirring. Due to the reactivity of this precursor solution to ambient humidity, it was transferred to the reaction flask using a sealed syringe. The reaction was then heated to 120 °C as fast as possible using a heating mantle and kept at 120 °C for 10 min. After the reaction period, the flask was cooled back to room temperature using a water bath. The NCs were precipitated from the crude solution with acetone (12 mL). After precipitation, the turbid solution was centrifuged at 12.1k rpm (20,130 g) for 1 min, and the supernatant was discarded. The NCs were washed by dispersion in *n*-butanol (1 mL) and precipitation with diethyl ether (20–40 mL depending on the colloidal stability), followed by centrifugation at 12.1k rpm (20,130 g) for 1 min. The product was finally dispersed in 2–6 mL of an alcohol (ethanol, isopropanol, or *n*-butanol) and centrifuged once more at 12.1k rpm (20,130 g) for 2 min to remove any aggregated particles.

## Supporting Information Available

Materials information, synthesis procedures, characterization details, analysis of an alumina sol, SEM EDX data, PXRD pattern, ζ potential measurements, ICP–MS data of alumina-gel-coated NCs, coating thickness and sizing curve calculations, TEM images of different sizes and different compositions, as well as additional TRPL measurements (PDF)

## Notes

The datasets supporting this article are made available through Zenodo at https://doi.org./10.5281/zenodo.7645376.
