# Supplementary material for: Colloidal CsPbX3 Nanocrystals with Thin Metal Oxide Gel Coatings
Source: Chem Mater. 2023 Mar 20;35(7):2827–34. doi: 10.1021/acs.chemmater.2c03562 (PMC10100534; doi:10.1021/acs.chemmater.2c03562)
Supplement: Supplementary file 1 — cm2c03562_si_001.pdf [file cm2c03562_si_001.pdf]

Supporting Information for:

Colloidal $CsPbX_3$ Nanocrystals with Thin Metal Oxide Gel Coatings

Dominic Guggisberg,[a,b] Sergii Yakunin,[a,b] Christoph Neff,[a] Marcel Aebli,[a,b] Detlef Günther,[a] Maksym V. Kovalenko,[a,b,c]* Dmitry N. Dirin[a,b,c]*

[a]Institute of Inorganic Chemistry, Department of Chemistry and Applied Biosciences, ETH Zürich, Vladimir Prelog Weg 1, CH-8093 Zürich, Switzerland

[b]Laboratory for Thin Films and Photovoltaics, Empa − Swiss Federal Laboratories for Materials Science and Technology, Überlandstrasse 129, CH-8600 Dübendorf, Switzerland

[c]NCCR Catalysis, Institute of Inorganic Chemistry, Department of Chemistry and Applied Biosciences, ETH Zürich, Vladimir Prelog Weg 1, CH-8093 Zürich, Switzerland

Chemicals

The following reagents were used as received: lead (II) bromide ($PbBr_2$, 98 %, abcr), trioctylphosphine oxide (TOPO, ~90 %, Strem), octane (Roth), hexane (Sigma-Aldrich), cesium carbonate (Cs_2CO_3, >99 %, Sigma-Aldrich), diisooctylphosphinic acid (DOPA, ~90 %, Sigma-Aldrich), 3-(N,N-dimethyloctadecyl-ammonio)propanesulfonate (ASC18, >99 %, Sigma-Aldrich), ethanol (>99 %, Sigma-Aldrich), toluene (Sigma-Aldrich), ethyl acetate (Sigma-Aldrich), oleic acid (OA, 90 %, Sigma-Aldrich), 1-octadecene (ODE, Sigma-Aldrich), lead (II) acetate trihydrate ($Pb(OAc)_2$*$3H_2O$, >99 %, Sigma-Aldrich), trioctylphosphine (TOP, >97 %, Strem), mesitylene (Sigma-Aldrich), bromine (Br_2, Acros), aluminum chloride ($AlCl_3$, 99.99 %, abcr), aluminum bromide ($AlBr_3$, 99 %, abcr), aluminum iodide (AlI_3, 99.99 %, abcr), aluminum ethoxide ($Al(OEt)_3$, 97 %, Sigma-Aldrich), aluminum sec-butoxide ($Al(O^sBu)_3$, >95 %, Fluka), aluminum tert-butoxide ($Al(O^tBu)_3$, 97 %, Alfa Aesar), aluminum phenoxide ($Al(OPh)_3$, 99.9 %, Sigma-Aldrich), acetone (Sigma-Aldrich), butanol (Sigma-Aldrich), diethyl ether (Sigma-Aldrich), isopropanol (Sigma-Aldrich), zirconium bromide ($ZrBr_4$, 98 %, abcr), zirconium butoxide ($Zr(OBu)_4$, 80 % in n-butanol, abcr), titanium bromide ($TiBr_4$, 98 %, Acros), titanium isopropoxide ($Ti(O^iPr)_4$, 97 %, Fluka)

Synthesis

Two types of synthesis were used for the starting $CsPbX_3$ NCs capped with organic ligands, with the TOPO-based room temperature synthesis giving more spherical NCs and the hot-injection synthesis giving cuboidal NCs. The optical properties (absorption, emission, QY) of NCs obtained from either method were very similar with NCs synthesized at room temperature being slightly more monodisperse. Therefore, both types were used interchangeably in this publication. The most significant difference was that the hot-injection method yielded 10-50 times higher amounts of NCs in one synthesis. Therefore, these NCs were used for comparative studies and screening experiments.

TOPO-based room temperature Synthesis:[1]

$PbBr_2$-TOPO (0.04 M): $PbBr_2$ (367 mg, 1 mmol) and TOPO (1.933 g, 5 mmol, ~90 %) were dissolved in 5 ml of octane at 120 °C in a 40 ml vial on a hot plate. Once all the $PbBr_2$ was dissolved (~30 min), the vial was cooled to room temperature and 20 ml of hexane was added. The stock solution was stored in air.

Cs-DOPA (0.02 M): Cs_2CO_3 (100 mg, 0.3 mmol) and DOPA (1 mL, 3.75 mmol) were mixed with octane (2 mL) at 120 °C in a 40 ml vial. Once all the Cs_2CO_3 was dissolved (~20 min), the stock solution was cooled to room temperature and 27 ml of hexane was added. The stock solution was stored in air.

ASC18 (0.05 M) stock solution: ASC18 (420 mg, 1 mmol) was added to butanol (20 mL). Due to not being fully soluble, the solution had to be heated to 50 °C prior to use.

Zwitterion capped $CsPbBr_3$ NCs: A previously reported procedure was upscaled and slightly modified for this synthesis.[1] Hexane (30 ml) was mixed with the $PbBr_2$ stock solution (5 ml) in a 100 mL beaker. Under heavy stirring, the Cs-DOPA stock solution (2.5 ml) was injected. After 8 minutes of growth the ASC18 solution (4 mL) was added to the crude $CsPbBr_3$ NC solution. After 1 minute, the solution was concentrated to ~4 ml on a rotary evaporator (70 mbar) at room temperature. The concentrated crude solution was centrifuged at 12.1k rpm (20130 g) for 1 minute which removed the excess ASC18 that is not soluble in apolar solvents. In order to improve the NC yield, the gel-like ASC18 was extracted twice with 4 mL toluene and centrifuged each time. The combined NC solutions were precipitated with ethyl acetate (36 mL) and centrifuged at 12.1k rpm (20130 g) for 1 min. The supernatant was discarded and the precipitate was dispersed in 4 mL toluene. The NCs were washed two more times by precipitation with 12 mL ethyl acetate, centrifugation and dispersion in 12 mL toluene. After the last dispersion the solution was once more centrifuged at 12.1k rpm (20130 g) for 2 min to remove aggregated particles.

The concentration of the final solution was determined from the absorption spectra using the absorption coefficient reported by Maes *et al*.[2]

Hot Injection Synthesis:[3]
Cs-oleate (0.4 M): Cs_2CO_3 (1.628 g, 5 mmol), OA (5 mL, 16 mmol) and ODE (20 mL) were heated to 120 °C under vacuum until no further gas evolution was observed.

Pb-oleate (0.5 M): $Pb(OAc)_2*3H_2O$ (4.58 g, 12 mmol), OA (8 mL, 25.3 mmol) and ODE (16 mL) were heated to 120 °C under vacuum until no further gas evolution was observed.

TOP-Br_2 (0.5 M): TOP (6 mL, 13.5 mmol) taken from the glovebox and reagent grade mesitylene (18 mL) were mixed in a capped 40 mL vial. Elemental bromine (0.6 mL, 11.6 mmol) was carefully added under vigorous stirring while controlling the temperature with a water bath.

Zwitterion capped $CsPbBr_3$ NCs: A previously reported procedure was upscaled for this synthesis.[3] Pb-oleate (5 mL, 0.5 M, 2.5 mmol), Cs-oleate (4 mL, 0.4 M, 1.6 mmol), ASC18 (201 mg, 0.48 mmol) and ODE (10 mL) were added to a 50 mL three-necked-flask and heated to 100 °C under vacuum. Once the gas evolution stopped, the flask was put under an inert atmosphere and the temperature was adjusted to 130 °C. After reaching the reaction temperature, TOP-Br_2 (5 mL, 0.5 M, 2.5 mmol) was injected and the reaction was cooled to room temperature immediately using an ice-bath.

To the crude solution 3.5 eq. ethyl acetate were added and the solution was centrifuged at 12.1k rpm (20130 g) for 1 min. The supernatant was discarded and the precipitate dispersed in 12 mL toluene. The NCs were washed two more times by precipitation with 36 mL ethyl acetate, centrifugation and dispersion in 12 mL toluene. After the last dispersion the solution was once more centrifuged at 12.1k rpm (20130 g) for 2 min to remove aggregated particles.

The concentration of the final solution was determined from the absorption spectra using the absorption coefficient reported by Maes *et al*.[2]

Metal oxide sol-gel coatings:
Alumina gel coated $CsPbBr_3$ NCs: In a typical synthesis 0.12 mmol of the ASC18-capped NCs were mixed with ODE (12 mL) and the toluene was evaporated under vacuum. In the glovebox a previously prepared solution of $Al(O^sBu)_3$ (0.36 mL, 0.5 M in mesitylene, 0.18 mmol, 1.5 eq.) was taken and mixed with a solution of $AlBr_3$ (48 mg, 0.18 mmol, 1.5 eq.) in 0.4 mL anhydrous mesitylene. This results in

roughly 0.8 mL of $Al_2Br_3(O^sBu)_3$ precursor solution which is injected into the NC solution at room temperature under continuous stirring. Due to the reactivity of this precursor solution to ambient humidity, it was transferred to the reaction flask using a sealed syringe. The reaction was then heated to 120 °C as fast as possible using a heating mantle and kept at 120 °C for 10 min. After the reaction period, the flask was cooled back to room temperature using a water bath. The NCs were precipitated from the crude solution with acetone (12 mL). After precipitation the turbid solution was centrifuged at 12.1k rpm (20130 g) for 1 min and the supernatant discarded. The NCs were washed by dispersion in n-butanol (1 mL) and precipitation with diethyl ether (20-40 mL depending on the colloidal stability) followed by centrifugation at 12.1k rpm (20130 g) for 1 min. This washing step can be repeated any number of times and will give a clean colloid (insignificant leftover of the alumina gel in solution as evidenced by ICP-MS) after 3-5 steps. The product was finally dispersed in 2-6 mL of an alcohol (ethanol, isopropanol or n-butanol) and centrifuged once more at 12.1k rpm (20130 g) for 2 min to remove any aggregated particles.

Alumina gel coating of different compositions: The protocol remains the same as described above, but any mixed Cl-Br or Br-I composition can be used instead of pure $CsPbBr_3$ NCs and $AlCl_3$ or AlI_3 be used instead of $AlBr_3$. The final composition (and therefore color) will be the stoichiometric mix of the used NCs and the aluminum halide. In order to obtain the intended emission color of the sol-gel coated NCs, the halide composition of the organically capped NCs had to be taken into account when calculating the aluminum halide ratios for the coating. This also means, that one type of halide cannot fully be exchanged for another halide.

Zirconia and Titania coatings: The synthetic protocol as well as the washing procedure remained the same as for the alumina-gel-coated NCs. For zirconia coatings $ZrBr_4$ and $Zr(OBu)_4$ were used, for titania coatings $TiBr_4$ and $Ti(O^iPr)_4$ were used.

Characterization

Absorption spectra: Optical characterizations were performed at ambient conditions. UV-Vis absorption spectra of colloidal NCs were collected using a Jasco V670 spectrometer in transmission mode. The NC concentrations were determined from the absorption spectra using the absorption coefficient reported by Maes *et al.*[2] For the measurements NCs solutions were diluted down to 20-50 µg/mL. Zwitterion-capped NCs were dispersed in either hexane or toluene. Metal-oxogel-coated NCs were dispersed in butanol.

Photoluminescence (PL): A Fluorolog iHR 320 Horiba Jobin Yvon spectrofluorometer equipped with a PMT detector was used to acquire steady-state PL spectra. NC solutions were measured in the same dilutions and solvents as the absorption measurements. NC films were obtained by spincoating concentrated solutions (≈10 mg/mL) and then measured in a 90° geometry.

Photoluminescence quantum yield (PLQY): Absolute PL QYs of films and solutions were measured with a Hamamatsu C13534 Quantaurus-QY Plus UV-NIR absolute PL quantum yield spectrometer. The same solutions and films that were used to measure PL were also used to measure QY.

Time-resolved photoluminescence (TRPL): Time-resolved PL traces were acquired in solution using a FluoTime300 spectrometer from PicoQuant.

Electron microscopy: TEM images were collected using a JEOL JEM-1400 Plus operated at 120 kV. SEM EDX was done on a FEI Quanta 200F equipped with an Octane Elect Super EDS system and was operated at 30 kV. NCs were deposited on carbon coated copper TEM grids from dilute solutions by drop casting.

X-Ray diffraction (XRD): XRD patterns were collected with a STOE STADI P powder diffractometer operating in transmission mode. A germanium monochromator, Cu Kα irradiation (λ = 1.540598 Å) and

a silicon strip detector (Dectris Mythen) were used. In order to measure an XRD pattern of NCs they were precipitated and dried to a powder which was then put between two stripes of Scotch tape.

Zeta potential (ZP): ZP measurements were performed on a Malvern Zetasizer Nano ZS. Ethanol absolute or anhydrous butanol were used as solvent, with crude NC solutions being diluted 100-300 times (to roughly 0.1 mg/mL) and the voltage being fixed to 10 V. The measured mobility data was automatically converted to zeta potential using Smoluchowski's theory and then fitted with a Gaussian function to obtain the average zeta potential.

NMR spectroscopy: ^{27}Al solid-state Magic Angle Spinning (MAS) NMR was measured on a 16.4 T Bruker Avance III HD spectrometer (Bruker Biospin, Fällanden, Switzerland). The instrument was equipped with a 2.5 mm double-channel solid-state probe head. The spinning frequency was set to 20 kHz. Chemical shifts were referenced to $Al(NO_3)_3$ in D_2O (1.1 M).
For the 1D spectra a full echo experiment was used with an excitation pulse of 3 µs. The echo delay was set to 40 cycles. 8192 transients were acquired with a recycle delay of 1 s.
The 2D multi quantum MAS (MQMAS) spectra were acquired using a Bruker 3Q MAS pulse program for odd half integer spin nuclei, using 3 pulses with full echo acquisition (mp3qdfs). The 90-degree hard pulse was set to 3.5 µs and the selective refocusing pulse to 40 µs. 2048 transients were acquired. The echo build-up time was set to 15 rotor cycles to detect a full echo.

ICP-MS: The elemental composition was measured on an ICP-QMS (Agilent 7500cs, United States). A microwave digestion approach including 20-30 mg sample, 1 ml HNO_3, 0.2 ml cobalt solution (digestion recovery control) was used. Indium was used as an internal standard. Three subsets of each sample were digested. Each subset was measured 3 times. External calibrations including Al, Co, Cs, Pb and In as an internal standard were used. The confidence intervals were calculated based on nine measurements with a confidence level of 95%.

Measurements

Additional TEM pictures:

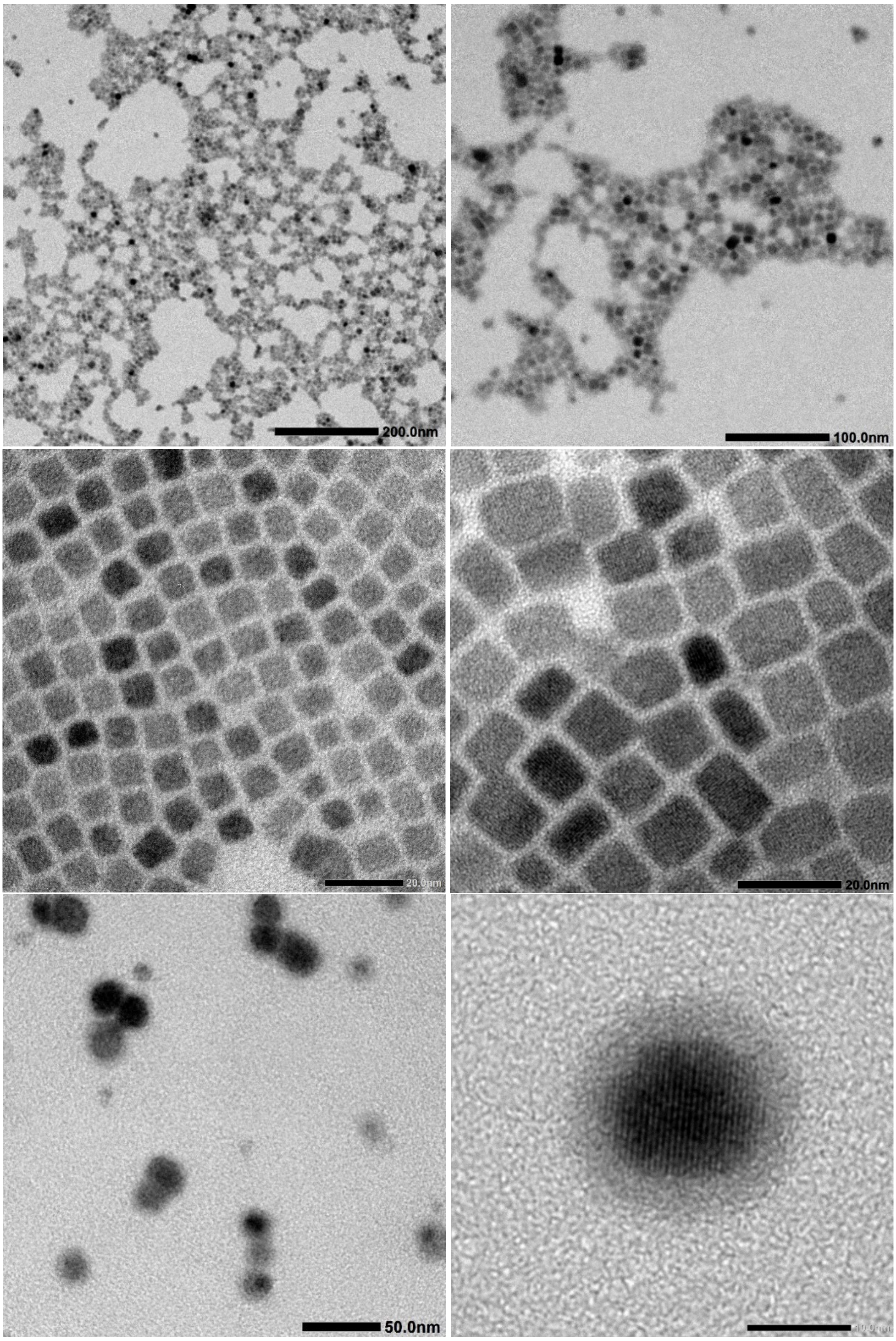

Figure S1: Top row: Overview TEM images of two well washed alumina-gel-coated NC samples. Middle row: High-resolution TEM pictures of two well washed samples, where the alumina gel coating is barely visible. Bottom row: High-resolution TEM pictures of two samples that were only washed once and the alumina gel coating was clearly visible.

Alumina Gel:

Pure alumina gel was synthesized by reacting $AlBr_3$ and $Al(O^sBu)_3$ in ODE at 120 °C for 10 min. The product was then precipitated with acetone and dispersed in butanol. The product was deliberately not washed further in order to keep as much of the gel as possible.

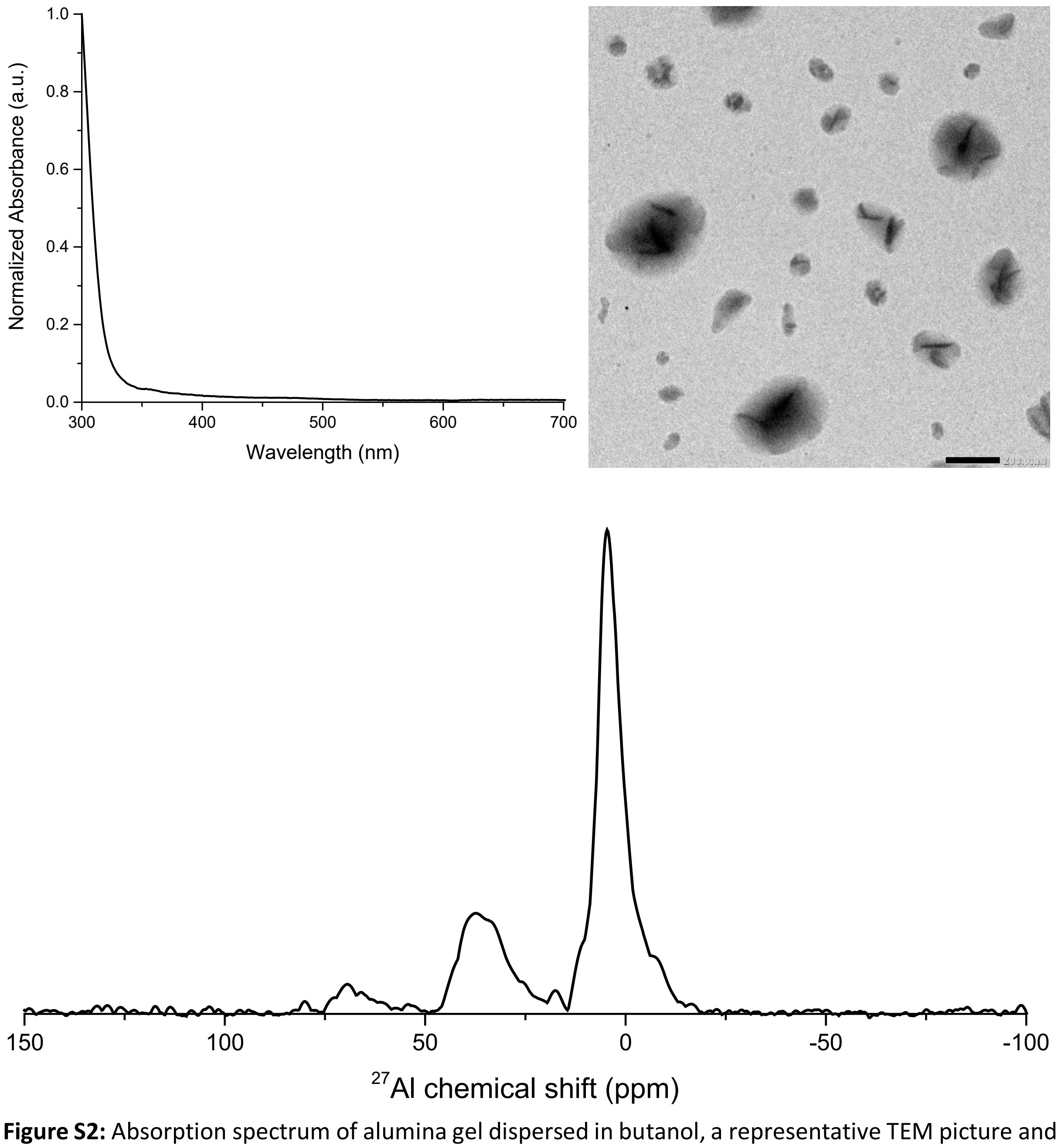

Figure S2: Absorption spectrum of alumina gel dispersed in butanol, a representative TEM picture and the respective ^{27}Al NMR spectrum with 4-, 5- and 6- fold coordinated AlO_x-species visible.[4]

PXRD pattern of alumina-gel-coated LHP NCs:

Besides being very thin the coating is also inherently amorphous due to being a gel. To confirm this, we measured powder X-ray diffraction of coated NCs which were not washed so that the most amount of alumina gel remains in the sample. The obtained diffractogram clearly shows the orthorhombic *Pnma* structure of the perovskite NCs[5] but no peaks appear for the alumina gel.[4]

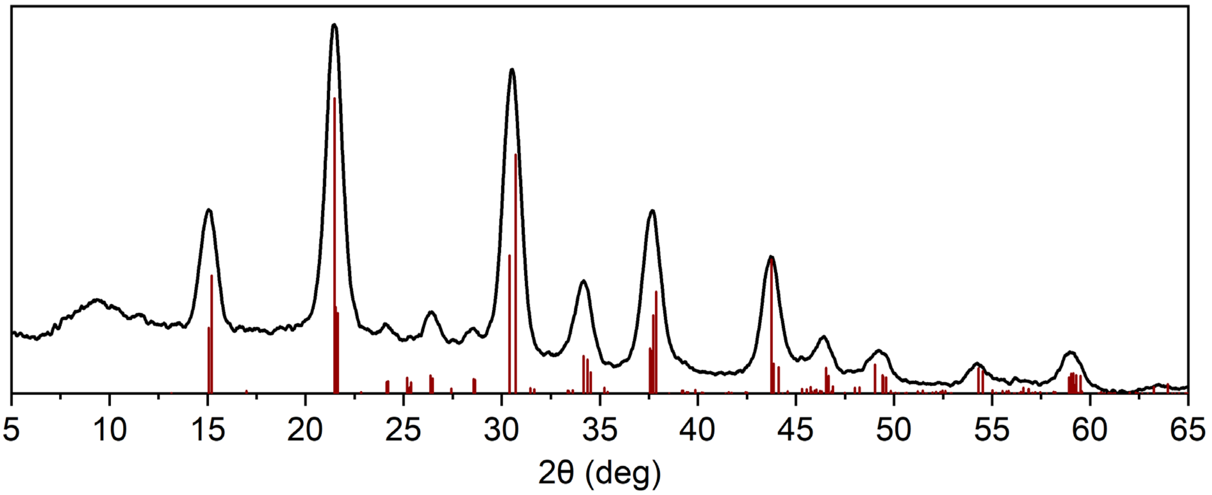

Figure S3: XRD pattern of a typical alumina-gel-coated $CsPbBr_3$ NC sample, matching orthorhombic (*Pnma*) $CsPbBr_3$ (red lines)[5] but showing no signs of any other crystalline component.[4]

SEM EDX:

Another well-established tool for elemental analysis is energy-dispersive X-ray spectroscopy (EDX). SEM EDX was performed on a well washed sample in an attempt to determine the elemental composition of intact NCs. Unfortunately, the characteristic X-rays for the Al Kα overlap with the Br Lα so closely that they become indistinguishable (see Fig. S4). Comparing the integrated Br Lα and Br Kα ratios of pristine and coated NCs could in principle be used to determine the amount of Al. However, the 29 % increase in signal intensity of alumina gel coated compared to pristine NCs was not deemed significant enough over the inherent variance in signal intensity for low energy X-rays to be used as a proof for the presence of Al.

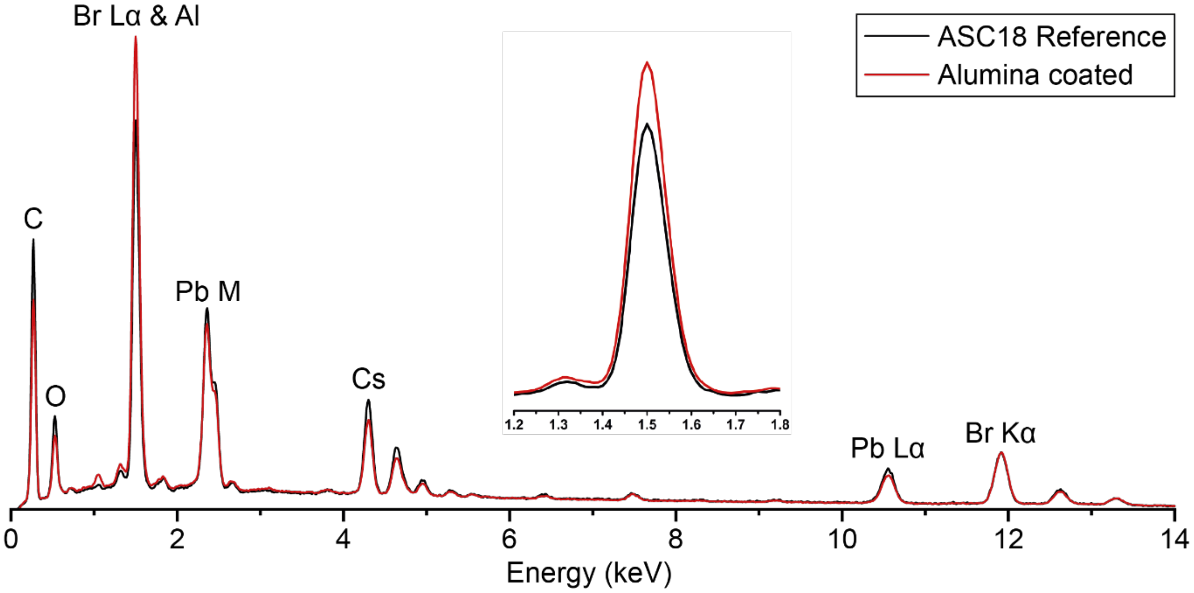

Figure S4: SEM EDX comparison of ASC18 capped NCs (black) and alumina-gel-coated NCs (red). The spectra are normalized to the Br Kα peak and the inset shows a zoomed in cutout of the combined Br Lα and Al peak.

ICP-MS:

Table S1: Elemental analysis results for Al, Cs and Pb given in mass% of the whole sample. Sample 1 was NCs that were washed 5 times, leaving insignificant amounts of alumina gel left in solution. Sample 2 was the leftover alumina gel that was washed away from sample 1. Sample 3 was washed once and sample 4 was not washed.

**Sample**	**Al [%]**	**Cs [%]**	**Pb [%]**	**Cs:Pb**	**Al:Pb**
1	1.85 ± 0.07	16.78 ± 0.10	26.4 ± 0.3	0.99	0.54
2	8.22 ± 0.17	7.30 ± 0.04	10.84 ± 0.11	1.05	5.82
3	4.09 ± 0.20	13.36 ± 0.05	20.88 ± 0.24	1.00	1.50
4	6.25 ± 0.10	9.92 ± 0.03	15.44 ± 0.12	1.00	3.11

Coating Thickness calculations:

The ICP-MS results gave us a ratio of Al to Pb (and Cs) and assuming that in the case of the well washed NCs all Al is located on the surface of the NCs in form of a coating, we can calculate the thickness of this coating. For the sake of this calculation we are additionally assuming that we are dealing with perfect cubes, although analogous calculation for the spherical NCs would lead to the exact same result. The calculation uses the volume of a cube and a coated cube as well as the density of the two materials to calculate the coating thickness.

The ratio r of Al to Pb can be expressed as the molar ratio of the two compounds with a correction factor of 2 to accommodate for the 2 Al in Al_2O_3 and n being the molar amount.

$$r = \frac{2n_{Al}}{n_{Pb}} \tag{1}$$

The molar amounts can be substituted with the molecular weight M and the mass m using equation (2) which leads to equation (3).

$$n = \frac{m}{M} \tag{2}$$

$$r = 2\frac{m_{Al} \times M_{Pb}}{m_{Pb} \times M_{Al}} \tag{3}$$

The mass can be substituted with the volume V and the density δ using equation (4).

$$m = V \times \delta \tag{4}$$

$$r = 2\frac{V_{Al} \times \delta_{Al} \times M_{Pb}}{V_{Pb} \times \delta_{Pb} \times M_{Al}} \tag{5}$$

For the two volumes we can look at a cube with diameter d and a coating thickness s. The volume of the perovskite V_P is given in equation (6) and the volume of the alumina gel coating V_{Al} is given in equation (7). Putting these into equation (5) leads to equation (8)

$$V_P = d^3 \tag{6}$$

$$V_{Al} = (d + 2s)^3 - d^3 \tag{7}$$

$$r = 2\frac{((d+2s)^3 - d^3) \times \delta_{Al} \times M_{Pb}}{d^3 \times \delta_{Pb} \times M_{Al}} \tag{8}$$

For clarity we pulled all constants together and made a factor $a = 2\frac{M_P}{\delta_P \times M_{Al}}$. The density of the coating was purposely not put into the factor so it could easily be changed afterwards for estimations on coatings with lower density. Using $M_{Pb} = 579.82\ g/mol,\ M_{Al} = 101.96\ g/mol$ and $\delta_{Pb} = 4.8\ g/cm^3$ the factor a equals to $a = 2.369\ cm^3/g$. Equation (8) was then rearranged for the coating thickness s giving (9).

$$s = d\frac{\sqrt[3]{a^2\times\delta_{Al}{}^2\times(a\times\delta_{Al}+r)}-a\times\delta_{Al}}{2\times a\times\delta_{Al}} \quad (9)$$

Since the density of the alumina sol-gel is unknown, we used the numbers of Al_2O_3 ($\delta_{Al2O3} = 3.96\ g/cm^3$) and $Al(O^sBu)_3$ ($\delta_{Al(OsBu)3} = 0.967\ g/cm^3$) as the lower and upper limit for the coating thickness (upper and lower limit for the coating density respectively). With a NC size of 10 nm, a ratio r of 0.5 would only give a coating thickness in the range of $s = 0.09 - 0.33\ nm$.

Zeta Potential measurements of alumina-gel-coated LHP NCs:

Electrophoretic mobility measurements were conducted on the solutions of NCs diluted to 0.1 mg/mL in ethanol or butanol, at 8-12 V. The reference measurement cannot be performed for the alumina gel itself due to the uncontrolled size of the gel species. The closest analogy to the alumina-gel-coated NCs would be alumina nanoparticles since the charges originate from the same surface-adsorbed hydroxo species in both cases. Reported zeta potentials for alumina nano-/microparticles at pH 7 range from +20 to +50 mV which matches with the measured data.[6-8]

Table S2: Zeta potential measurements of $CsPbBr_3$/alumina gel samples with different amounts of NEt_3.

Eq. of NEt_3	ZP [mV]
0	+37.6
0	+38.8
0	+36.1
0	+31.3
0	+35.5
0	+33.9
0	+34.1
0	+37.2
2.5	+31.4
2.5	+26.1
5	+13.7
5	+12.9
12.5	+9.02
12.5	+8.28
25	+5.85
25	+3.99
62.5	+3.98
62.5	+3.22
125	+2.32
125	+1.21
250	-0.19
250	-1.48
500	-1.31
500	-2.97
750	-1.79
750	-3.38

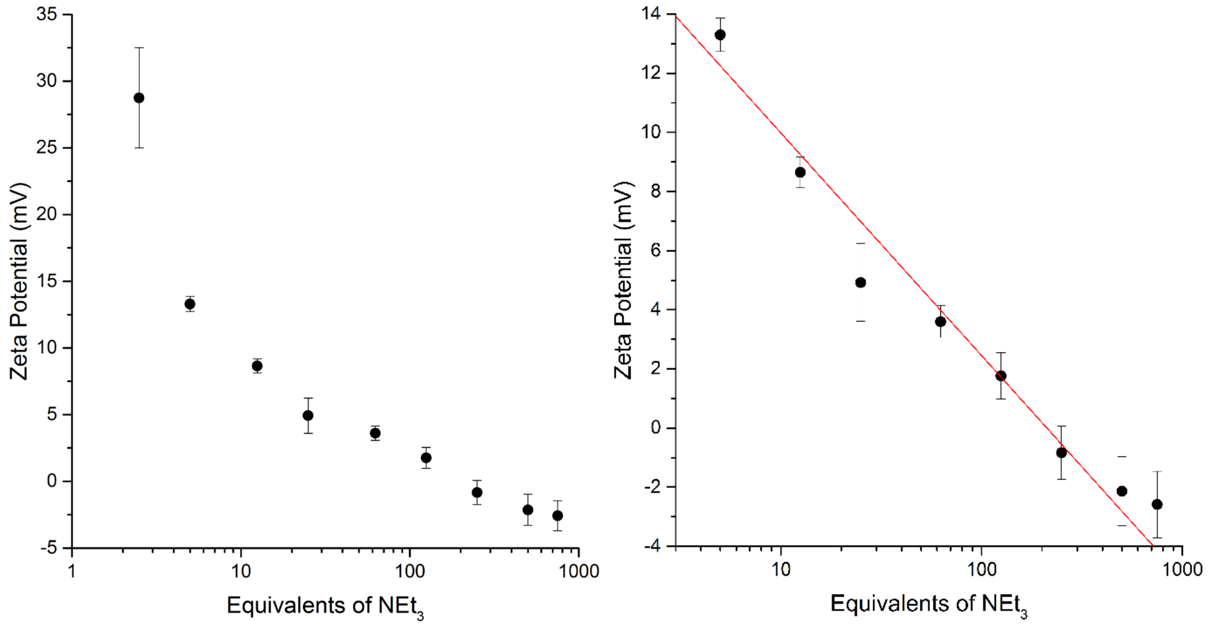

Figure S5: Zeta potential measurements plotted with a logarithmic scale, showing that over the measured range the zeta potential decreases linearly with the logarithm of the amount of base added. This is similar to the linear dependence of the zeta potential with pH that is often reported for aqueous solutions. The first point at 2.5 equivalents does not follow the trend, presumably because the amount added is so low, that its effect is basically quenched.

Alumina-gel-coated NCs of different sizes:

5.3 nm: 8.5 nm: 13.6 nm:

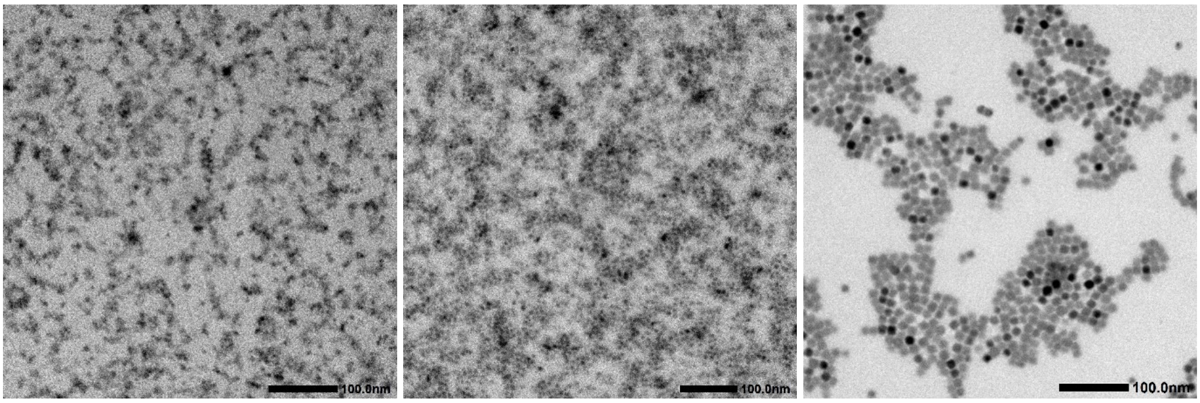

Figure S6: TEM pictures of alumina-gel-coated NCs of different sizes.

Sizing curve and calculation:

The average NC size s (in nm) of a sample was determined from the maximum of the first excitonic peak a (in eV) in the absorbance spectra using the sizing curve and formula reported by Akkerman *et al.*[1]

$$s = 1.904 \times (a - 2.381)^{-0.589} \qquad (10)$$

To get the correct maximum of the first excitonic peak the absorbance spectra were converted to energy scale and the second derivative was calculated. This eliminates the inherent underlying rise in absorbance and the possible scattering that would obscure the correct maximum.

Alumina-gel-coated NCs of different compositions:

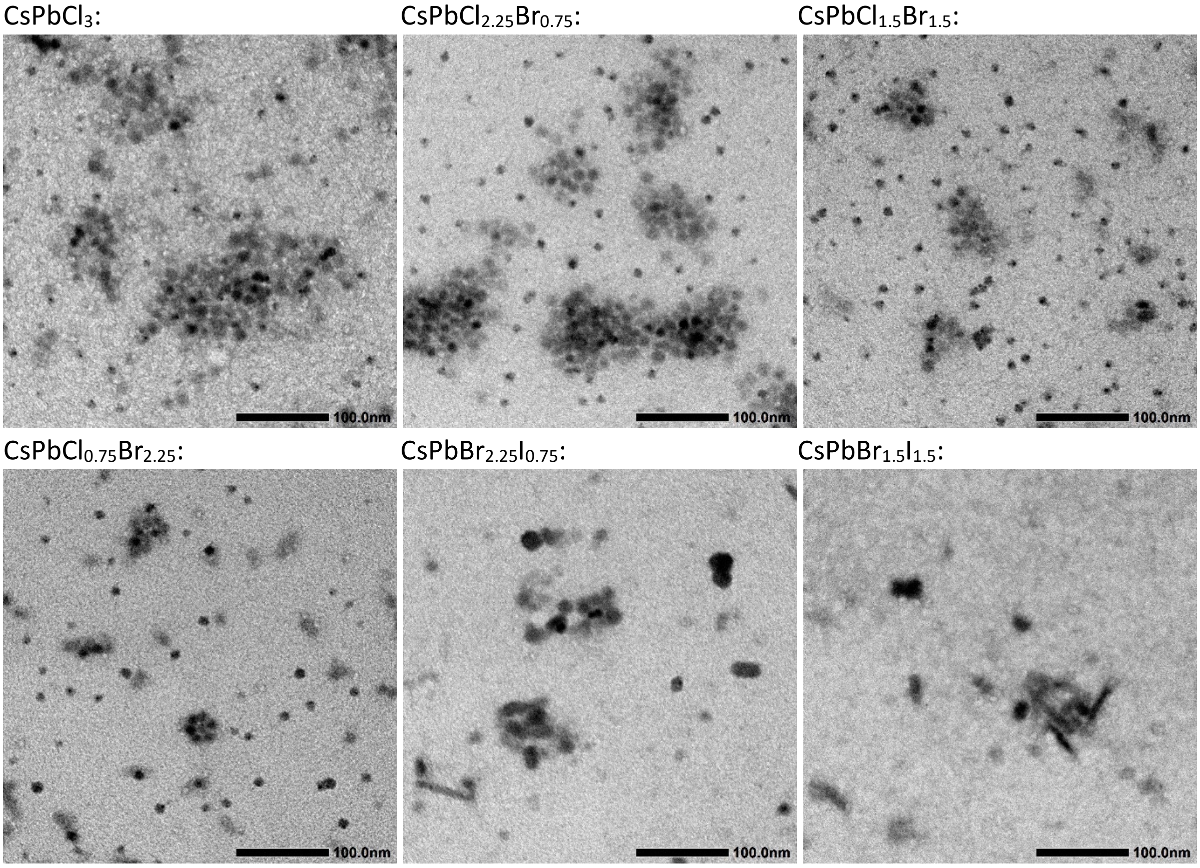

Figure S7: TEM pictures of alumina-gel-coated NCs with different compositions.

Humidity effects on emission properties of alumina-gel-coated NCs in compact films:

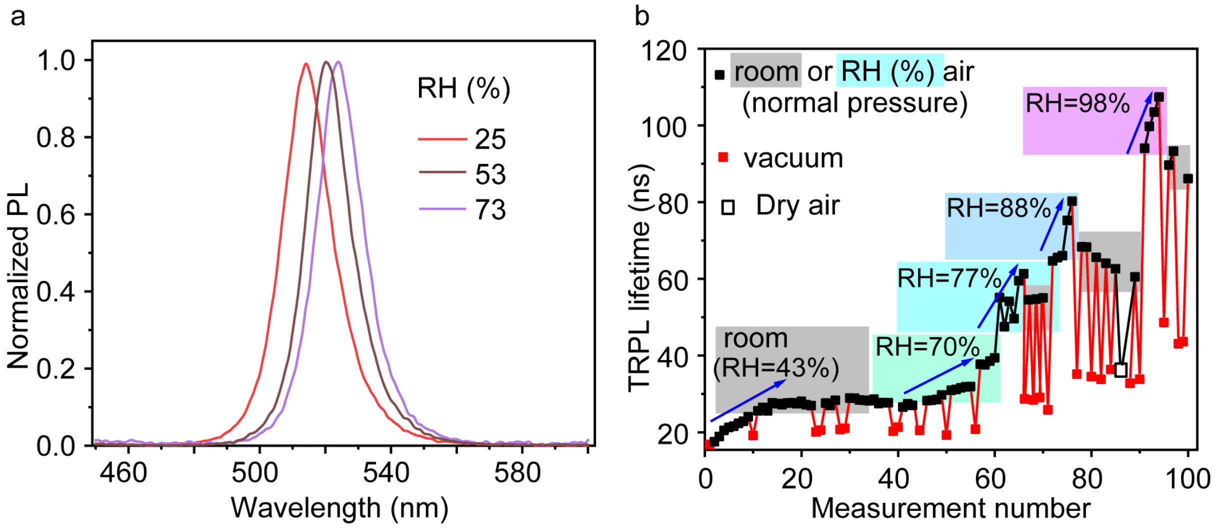

Figure S8: (a) PL emission spectra shift under variation of the humidity conditions. (b) Evolution of the PL lifetime with gradual increase of humidity (filled black dots) with relaxation to ambient room humidity (RH = 43 %, gray areas) and cycling to the vacuum (red dots) and dry air (open black dot) conditions.

Humidity effects on PLQY of alumina-gel-coated NCs in compact films:

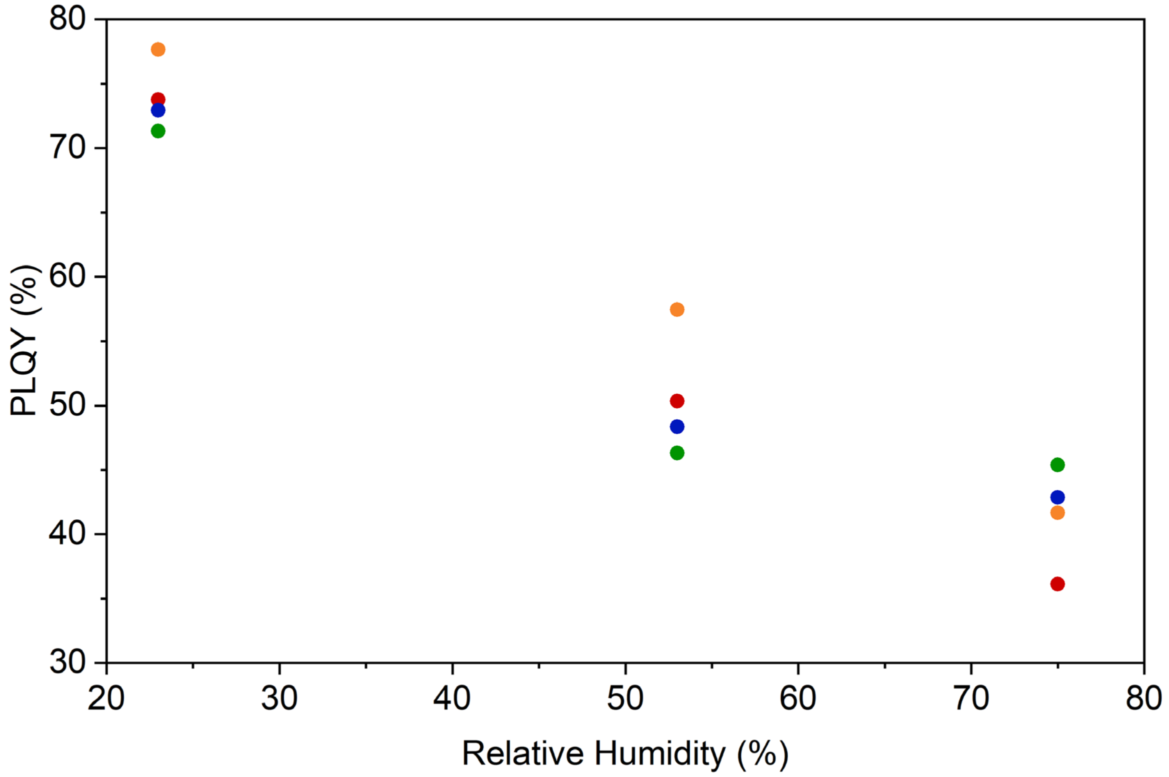

Figure S9: PLQY of four alumina-gel-coated $CsPbBr_3$ samples measured at 3 different relative humidities (23 %, 53 % and 75 %).

References

1. Akkerman, Q. A.; Nguyen, T. P. T.; Boehme, S. C.; Montanarella, F.; Dirin, D. N.; Wechsler, P.; Beiglböck, F.; Rainò, G.; Erni, R.; Katan, C.; Even, J.; Kovalenko, M. V., Controlling the nucleation and growth kinetics of lead halide perovskite quantum dots. *Science* **2022,** *377* (6613), 1406-1412.

2. Maes, J.; Balcaen, L.; Drijvers, E.; Zhao, Q.; De Roo, J.; Vantomme, A.; Vanhaecke, F.; Geiregat, P.; Hens, Z., Light Absorption Coefficient of CsPbBr3 Perovskite Nanocrystals. *The Journal of Physical Chemistry Letters* **2018,** *9* (11), 3093-3097.

3. Krieg, F.; Ochsenbein, S. T.; Yakunin, S.; ten Brinck, S.; Aellen, P.; Süess, A.; Clerc, B.; Guggisberg, D.; Nazarenko, O.; Shynkarenko, Y.; Kumar, S.; Shih, C.-J.; Infante, I.; Kovalenko, M. V., Colloidal CsPbX3 (X = Cl, Br, I) Nanocrystals 2.0: Zwitterionic Capping Ligands for Improved Durability and Stability. *ACS Energy Letters* **2018,** *3* (3), 641-646.

4. Acosta, S.; Corriu, R. J. P.; Leclercq, D.; Lefèvre, P.; Mutin, P. H.; Vioux, A., Preparation of alumina gels by a non-hydrolytic sol-gel processing method. *Journal of Non-Crystalline Solids* **1994,** *170* (3), 234-242.

5. Bertolotti, F.; Protesescu, L.; Kovalenko, M. V.; Yakunin, S.; Cervellino, A.; Billinge, S. J. L.; Terban, M. W.; Pedersen, J. S.; Masciocchi, N.; Guagliardi, A., Coherent Nanotwins and Dynamic Disorder in Cesium Lead Halide Perovskite Nanocrystals. *ACS Nano* **2017,** *11* (4), 3819-3831.

6. Sprycha, R., Electrical double layer at alumina/electrolyte interface: I. Surface charge and zeta potential. *Journal of Colloid and Interface Science* **1989,** *127* (1), 1-11.

7. Singh, B. P.; Menchavez, R.; Takai, C.; Fuji, M.; Takahashi, M., Stability of dispersions of colloidal alumina particles in aqueous suspensions. *Journal of Colloid and Interface Science* **2005,** *291* (1), 181-186.

8. López Valdivieso, A.; Reyes Bahena, J. L.; Song, S.; Herrera Urbina, R., Temperature effect on the zeta potential and fluoride adsorption at the α-Al2O3/aqueous solution interface. *Journal of Colloid and Interface Science* **2006,** *298* (1), 1-5.
